# How Psychological Resilience Mediates the Effect of Caregiver Frustration on the Health of Caregivers

**DOI:** 10.1093/geroni/igaf122.3957

**Published:** 2025-12-31

**Authors:** Victor Elisha, Emil Coman, Richard Fortinsky

**Affiliations:** University of Connecticut, Farmington, Connecticut, United States; UConn Health Disparities Institute, Farmington, Connecticut, United States; University of Connecticut, Farmington, Connecticut, United States

## Abstract

Family caregivers are critically important in supporting older adults living with dementia. Caregiving responsibilities often lead to negative emotional responses including frustration, which could negatively affect caregivers’ health and well-being. However, adverse effects of caregiver frustration might be mitigated by psychological resilience, which is an established mediator in the manifestation of negative health outcomes in other populations. This cross-sectional study used path analysis to examine relationships between caregiver frustration, psychological resilience, and self-reported health in 291 caregivers of older adults living with dementia. The model hypothesized direct paths connecting caregiver frustration, psychological resilience and self-reported health and used maximum likelihood estimation to obtain path coefficients. Results revealed that increased caregiver frustration was associated with lower psychological resilience (β = -0.41: p < 0.0001, 95% CI [-0.59, -0.22]) and psychological resilience was positively associated with self-reported health (β = 0.32: p < 0.0001, 95% CI [0.17, 0.48]. The indirect effect of caregiver frustration on self-reported health via resilience was estimated as -0.13 (p = 0.003, 95% CI [-0.22, -0.05]), while the direct effect of caregiver frustration on self-reported health was not statistically significant (β = -0.083: p = 0.52, 95% CI [-0.34, -0.17]). We conclude from this pattern of results that the compromising effect of caregiver frustration on health outcomes among caregivers of people living with dementia occurs through diminished psychological resilience, highlighting the potential benefit of psychological resilience-building interventions in caregiver support programs.

